# An Automated Water Task to Test Visual Discrimination Performance, Adaptive Strategies and Stereotyped Choices in Freely Moving Mice

**DOI:** 10.3389/fnbeh.2018.00251

**Published:** 2018-11-08

**Authors:** Mario Treviño, Esteban Fregoso, Carlos Sahagún, Elí Lezama

**Affiliations:** Laboratorio de Plasticidad Cortical y Aprendizaje Perceptual, Instituto de Neurociencias, Universidad de Guadalajara, Guadalajara, Mexico

**Keywords:** mouse, visual discrimination, stereotypy assay, visual cortex (V1), entropy, pharmacological inactivation

## Abstract

We describe an automated training/testing system for adult mice that allows reliable quantification of visual discrimination capacities, adaptive swimming strategies, and stereotyped choices with minimal human intervention. The experimental apparatus consists of a hexagonal swimming pool with an internal decision zone leading to three interior arms with two software-controlled platforms inside of each arm. Each experimental trial consists in projecting a “positive” conditioned discriminative stimulus (S^D^) in one randomly chosen arm, whereas the other two arms project non-reinforced stimuli (the delta stimuli, S^Δ^). By employing a classical behavioral training schedule, the mice learn to swim toward the arm that displays the S^D^, because it predicts the presence of two elevated platforms located symmetrically to the left and right side of the projecting monitor. Separate behavioral components for discriminative and stereotyped choice behavior can be identified through this geometric arrangement. In addition, the projection in real-time of either static or dynamic visual stimuli allows the usage of training programs contingent on current behavioral performance. We validated the system by characterizing the visual acuity and contrast sensitivities in a group of trained mice. By employing pharmacological manipulations, we found that the mice required an intact functioning of the primary visual cortex (V1) to solve the hexagonal pool. Overall, the automated training system constitutes a reliable, rapid, and inexpensive method to quantify visual capacities of mice. It can be used to characterize visual and non-visual factors of choice behavior. It can also be combined with manipulations of visual experience and pharmacological micro-infusions to investigate integrated brain function and learning processes in adult mice over consecutive days.

## Introduction

Animal models are crucial to explore the cellular and circuit mechanisms involved in healthy and pathological brain function. Among these models, mice have become extremely valuable for neuroscience research due to their fast reproduction cycle, relatively small size and the vast amount of genetic and functional manipulations available. Several tasks have been developed to extract behavioral measures that characterize contextual (Wehner and Radcliffe, 2004), sensory (Prusky et al., 2000), locomotor (Keller et al., 2012; Saleem et al., 2013), and attentional/motivational processes (Bussey et al., 2008). Furthermore, mice have a broad repertoire of visually guided behaviors and are particularly noticeable for their learning and decision-making capacities (Busse et al., 2011; Glickfeld et al., 2013; Trevino et al., 2013). Tests of mouse vision are essential not only for exploring the mechanisms of vision itself, but also to study integrated brain functions that require a combination of perceptual learning, attentional and decision-making processes. Behavioral paradigms for assessing the visual performance of mice can be grouped into those that involve innate visual reflexes and those that require training. Optokinetic (eye movements) and optomotor (compensatory head movements) responses can be easily evoked by drifting the visual surround. These reflexes occur mainly because the vestibular input (the sense of balance and spatial orientation) is dissociated from the processing of visual information (Kretschmer et al., 2015). In contrast, the study of learned behavior under the control of visual stimuli involves training paradigms that allow the mice to associate a visual stimulus with reward (Trevino et al., 2011, 2013; Yu et al., 2018). Because rodents are good swimmers by nature, a common approach to train and test their visual function has been to adapt a two alternative forced choice discrimination task inside a trapezoidal water maze (Prusky et al., 2000; Trevino et al., 2012, 2013; Trevino, 2014). This task, however, has many critical disadvantages: (1) the shape of the pool and the spatial arrangement of the two arms allow the mice to have either focused or mixed access to both S^D^/S^Δ^ stimuli. This methodological uncertainty makes the implementation of decision-making models difficult, particularly of those based on mechanisms of mutual and/or feed-forward inhibition (Bogacz et al., 2006). (2) The polarized geometry of the maze that impedes the usage of powerful behavioral indexes that require distributed variances of the swimming trajectories (Maei et al., 2009). (3) The procedure of manually picking up the mice from the rewarding platform introduces excessive unwanted “dead times” which also create variable handling and additional stress sources to the procedure.

Besides these disadvantages, we must also consider that the ability of the mice to solve a water task depends on many factors. Mice implement diverse swimming strategies which can be thoroughly studied by analyzing the complexity of their swimming trajectories. Stereotyped choices constitute an example of an adaptive behavioral strategy that occurs regularly in psychophysical experiments (Prusky et al., 2000; Busse et al., 2011; Trevino et al., 2013; Trevino, 2014). Stereotypical behavior is a term used to describe a wide variety of invariant behaviors that could maximize utilities (Killeen et al., 2018) but that could also derive from inherent properties of individuals (Trevino, 2014). In mice, stereotyped choices are strongly dependent on reward and sensory histories, they are consistent across animals and determine their learning trajectories (Trevino, 2014; Akrami et al., 2018). For those reasons, it is crucial to design experimental assays that allow precise quantification of repetitive behavior. Such tasks should be instrumental in linking stereotyped behavior to the underlying physiological properties of individuals.

To address all of the methodological considerations mentioned above, we developed a fully automated system to train and test visual discrimination capacities, adaptive swimming strategies, and stereotyped choices in adult mice. The apparatus consists of a hexagonally shaped pool with a decision area in the center of the pool that allows access to three visual stimuli, one at a time, and with six non-visible computer-controlled escape platforms. We validated the system by training a group of mice until they reached a high visual discrimination performance. We then characterized their choice stereotypies, visual acuities, and contrast sensitivities. We also made pharmacological inactivations of the primary visual cortex (V1) of these mice and found that this manipulation strongly impaired their visual performance. Altogether, this automated task allows a rapid estimation, with strong statistical power, of the visual (discriminative) and non-visual (stereotypical) choice behavior of mice, and to quantify the error and path entropies of their swimming trajectories over consecutive trials.

## Methods

### Animals

We used 8-weeks-old C57BL/6J male mice (18–28 g) housed in groups of 2–3 mice in standard polycarbonate cages (Alternative Design, USA; 29.2 × 18.4 × 12.7 cm) under conventional laboratory conditions, with food (Rodent Lab Chow 5001, Purina) and water *ad libitum*. The housing room operated in a regular 12:12 h. light/dark cycle (lights on from 8:00 a.m. to 8:00 p.m.) with constant room temperature (22 ± 2°C) and humidity (55 ± 20%). The animals were trained and tested in the light phase of the day, between 8 a.m. and 2 p.m., from Monday to Friday, in groups of ten, each session consisting of max. 70 trials/day, lasting ~60–70 min. All animal experiments were carried out following the Mexican animal welfare guidelines (SAGARPA, NOM-062-ZOO-1999), in line with the NIH's Guide for the Care and Use of Laboratory Animals. The ethics committee of the “Instituto de Neurociencias,” Universidad de Guadalajara, México, approved our experimental protocol (ET062017-243).

### Apparatus

The training/testing apparatus consisted of a hexagonal glass pool filled with water. Each side of the hexagon measured 50 cm long, 50 cm height, and 0.9 cm thick, yielding a polygon circumscribed in an imaginary circle of 50 cm radius. Three white 3 mm thick acrylic dividers extended from the side walls toward the center of the pool, creating a decision chamber with access to three interior arms facing three computer-controlled monitors placed in front of non-adjacent sides of the pool. Three pairs of computer-controlled acrylic platforms (8 cm long, 8 cm wide, 18 cm high) were placed adjacent to the sides of the dividers. Each platform was controlled independently of the others and could adopt either a “submerged state” at 11 cm or an “elevated state” at 1 cm below the water surface, respectively. The pool was filled with 21°C ± 1°C tap water to a depth of 19.5 cm, generating a level of water 1 cm above the surface of the elevated platforms. The pool had a drain valve (1” = 2.54 cm) on a side wall and was placed on a solid square table (120 × 120 × 75 cm) in a quiet room destined for behavioral experiments.

#### Behavioral training and testing

The rationale of the task was to use the mouse's ability to associate a visual stimulus with escape from water (Trevino et al., 2013). The mice were released into the pool starting from one platform inside an arm (randomly chosen) and gradually learned to swim toward the S^D^ (correct choice) because they could reach one of the two elevated platforms and rest from swimming. Otherwise, by choosing the S^Δ^ (incorrect choice), the mice had to continue swimming until they found one of the elevated platforms in the S^D^ arm (Trevino et al., 2013). When choosing the “right arm” (projecting the S^D^), the mice were rewarded by being allowed to rest on the platform for 40 s, but they were allowed to rest only for 10 s when choosing the ‘wrong arm’. The selection of the arm displaying the S^D^ varied pseudo-randomly over trials, with the constraint that it could not appear on the same arm for more than one trial. All mice swam daily with a linearly increasing training regimen that went from 10 to 70 trials per day, improving their perceptual and physical performance in the task. Each session began by carefully placing a mouse onto one of the two elevated platforms (randomly chosen) from an arm projecting the S^D^. From this moment on, the automatic system took charge of performing the subsequent training trials. To test the visual acuity of the mice, we used static gratings with variable spatial frequencies at 100% contrast (in cycles/screen)|_repetitions_: 3|_10_, 9|_10_, 15|_14_, 20|_16_, 26|_16_. These values are equivalent to (in cycles/degree)|_repetitions_: 0.10|_10_, 0.29|_10_, 0.48|_14_, 0.64|_16_, 0.83|_16_ (Trevino et al., 2012). For contrast sensitivity experiments, we used static gratings of variable contrast with a low spatial frequency of three cycles/screen (CPS) in % contrast|_repetitions_: 0%|_28_, 12.5%|_16_, 25%|_10_, 37.5%|_10_, 100%|_2_ (Glickfeld et al., 2013). To eliminate gradients in average luminance between the screens that were projecting the S^D^/S^Δ^ stimuli, we restricted the spatial frequencies tested to full cycles (all stimuli had an average luminance of 235 ± 10 lux at 24 cm from the monitors, (Trevino et al., 2012, 2013). The experimenter was not visible to the mice during experiments. At the end of each training session, the animals were carefully dried with a towel and placed back in their home-cages. In colder weather, we recommend assisting the mice when they rest from swimming with external heating devices. The hexagonal pool was placed inside a quiet laboratory room without windows and lit with diffusely reflected light. We conducted all experiments in silence, with mobile phones switched off and in the absence of perfumes.

### Behavioral analysis

We assessed discrimination performance by calculating the percent of correct choices/mouse. We also recorded the animals' trajectories by using a computerized home-made video-tracking system, based on a web-camera (Microsoft LifeCam Studio; 30 FPS) fixed to the ceiling 190 cm above the bottom of the pool (Trevino et al., 2013). The video tracking algorithm located the position of a black mouse in a white background. We facilitated this process by placing the maze in a room with fixed intense illumination (~1,400 lux) and by setting up the acquisition properties of the camera to: 100% contrast, white-balance of 3,200, exposure of −7 (i.e., exposure time ≈7.81 ms), intermediate brightness of 175 and backlight-compensation turned on. We extracted multiple independent variables from the swim paths: path length (in cm), escape latency (i.e., time interval to reach the platform and climb it; in seconds), mean swimming speed (cm/s) and time spent in the S^D^ arm vs. the rest of the pool. We adapted an estimation of entropy (*H*) based on the sum of error entropy (i.e., the variance of the mouse's position relative to the target platform, *H*_*error*_) plus the path entropy (i.e., the variance of the mouse's position relative to the focus of its path, *H*_*path*_):

(1) $$\begin{array}{l} H=H_{error}+H_{path}=ln\;(\sigma_{d}^{2})+ln\;(\sigma_{a}\sigma_{b}) \end{array}$$

where σ_*d*_ corresponds to the distance of each point to the platform and σ_*a*_ and σ_*b*_ are the radii of each major axis of the error ellipse (Maei et al., 2009). Psychometric curves were estimated using non-linear methods (using the “lsqcurvefit” function from MATLAB) to fit the following logistic function to the observed averaged choices of the mice:

(2) $$\begin{array}{l} f\left(x\right)=\frac{L}{1+e^{-k\left(x-x_{0}\right)}} \end{array}$$

where *f(x)* is the probability of producing a correct choice at *x* spatial frequency or contrast, *L* is the curve's maximum value, *e* is the natural logarithm base, *k* is the slope and *x*_0_ is the *x*-value of the sigmoid's midpoint. Visual acuity and contrast sensitivity thresholds were defined as the value of the logistic fit at which the animal performed at 75% correct choices (Prusky and Douglas, 2004; Trevino et al., 2012). We report the visual acuity estimations both in cycles per screen and in cycles per degree, at 24 cm from the projecting monitors. To estimate the power-law exponent from sequential choice data, we calculated the absolute difference of the power spectra of the left and right sides of the choice sequences and plotted this difference against sequence length in a log-log plot (Trevino, 2014; Hanel et al., 2017). All algorithms were written in MATLAB R2014a (MathWorks, Inc.; Natick, USA). Visual stimuli were created and projected using the Psychophysics Toolbox extensions (PTB-3 (Brainard, 1997; Pelli, 1997).

### Surgical procedures

Before the surgeries, mice were placed in an isoflurane induction chamber (Sofloran, Laboratorios Pisa) with an increasing concentration of anesthetic (from 0.5 to 4%) over the course of 5 min. After isoflurane induction, the mice were anesthetized with Fentanyl (Fenodid, 0.15 mg/kg i.p.; Laboratorios Pisa), Midazolam (Dormicum, 6 mg/kg i.p.; Laboratorios Pisa) and Dexmedetomidine (Dexdomitor, 0.5 mg/kg i.p.; Orion Pharma). Their eyes were protected with ophthalmic lubricant (Eyelube; Hydroxypropyl Methylcellulose; Optixcare), and the incision points were pre-treated with small amounts of subcutaneous injections of lidocaine (Piscaína 2%; Laboratorios Pisa). After verifying that the level of anesthesia was suitable for surgery by using pinch and eye blink tests, we mounted the mice with non-rupture ear bars on a stereotaxic apparatus (Stoelting Co.; Model Nr.: 51730) and kept them at 37°C body temperature with a heating pad (Homeothermic Monitor; Harvard Apparatus, Model 50-7212). We shaved the scalp, cleaned it with a mixture of ethanol 70% and iodopovidone (Isodine, Boehringer) and made a midline incision. We cleaned the skull of all overlying connective tissue, scraped it and made the craniotomies with a dental drill (0.8 mm, Dremel 105; Foredom, MH-170). We used standard stereotaxic coordinates (Paxinos and Franklin, 2001) to bilaterally implant 30-gauge guide cannulae (made of stainless steel, BD PrecisionGlide™ Needles) targeting either the vibrissa motor cortex (VMC; 0.8 mm AP, 1 mm ML, 0.9 mm DV from the dura; Ebbesen et al., 2017) or the primary visual cortex (V1; −4.29 mm AP, 2.75 mm ML, 0.6 mm DV from the dura; Iurilli et al., 2012.) The tips of the cannulae were aimed 0.3 mm above the target structure, whereas the injector tips extended 0.3 mm beyond them. We also attached a head-fixation post (2 mm diam. × 8 mm length) to the skull with cyanoacrylate glue (Kola-Loka), fixed the cannulae and the post with dental acrylic cement (Nic-Tone R6V) and applied topical triple antibiotic (bacitracin 400 U/g, neomycin 3.5 mg/g, and polymyxin B 5 U/g; Polixín Ungena, Sophia). We inserted stainless steel obturators into the guide cannulae to prevent clogging. From this moment on, we housed the mice individually to avoid that they removed the obturators of other mice. To allow full recovery, we conducted the infusion experiments at least 5 days after surgeries.

### Drug application by micro-infusions

We habituated the animals for infusion handling by inserting 10% shorter dummy injectors into their cannulae 1 day before testing. To micro-infuse the animals, we lightly anesthetized them (isoflurane, 1–1.5%; Sofloran, Laboratorios Pisa), and removed the caps and obturators to insert the injectors. The injectors were connected via polyethylene tubing (0.75 mm internal diameter) to two independent 10 μl syringes (Hamilton, 701LT) simultaneously driven by a home-made microinfusion pump based on an Arduino board (Arduino, UNO R3) coupled to a motor-shield driver (DRV8825) to control a 1.8° Bi-polar stepper motor (NEMA 17HS8401). We injected either 500 nl or 1 μl of 25 mM muscimol (a GABA_A_ receptor agonist; Sigma-Aldrich, M1523) to inactivate V1 or the VMC, respectively. For some additional experiments in V1, we infused either a “low” (500 nl) or “high” (1,500 nl) volume of ibotenic acid (a toxin found in many mushroom varieties; Wood et al., 2018; 10 μg/μl; α-amino-3-hydroxy-5-isoxazoleacetic acid; Sigma-Aldrich, CAS # 2552-55-8), We prepared the drugs on the day of the infusion using NaCl 0.9% as the vehicle and infused during a maximum of 10 min at a rate of 0.1 μl/min (1.67 nl/s). We removed the injectors 5 min after finishing with the infusions (to allow the diffusion of the drug) and started the behavioral experiments 5 min later. The animals showed no signs of discomfort during or after injections.

### Whisking behavior

To quantify whisking behavior before and after injection of muscimol into the VMC, we filmed the snouts of head-fixed mice from above using a webcam (Microsoft LifeCam Studio; 1,920 × 1,080 pixels @ 30 FPS; Format: MP4). We selected two broad regions of interest (ROIs) covering all their left and right whisker sets and summed the pixel-by-pixel absolute difference of gray-scale transformed and adjusted images from consecutive video-frames (De Marco et al., 2014). Whisking power for each whisker set was calculated by computing the spectrogram of the whisker traces (500 samples at 30 FPS; noverlap = 0; nfft = 256) and then integrating the absolute value of the power spectral density from 0 to 15 Hz.

### Histology

To determine the location of the implanted cannulae used for pharmacological inactivation's, we euthanized the mice after completion of the behavioral experiments using deep anesthesia (sodium pentobarbital, Pisabental; Laboratorios Pisa) and fixed their brain via transcardial perfusion with 4% paraformaldehyde in 0.9% saline. We made coronal sections (50–100 μm) of the brains using a brain slicer (VT1000S, Leica). We mounted the sections on microscope slides, photographed them using a stereoscope (Stemi 305, Zeiss) and compared them against a reference atlas (Paxinos and Franklin, 2001).

### Statistical analysis

We compared choice and conditioned responses with one-sample *t*-tests and one-way ANOVA tests, psychometric curves with repeated measures ANOVA tests and cumulative distributions with Kolmogorov-Smirnov (KS) tests. All comparisons were followed by Bonferroni's or Wilcoxon Signed Rank *post hoc* tests. In **Figure 4D**, we tested the null hypothesis by creating 1,000 surrogates of the original data set, as previously described (Chamorro et al., 2017). We illustrate all our results as averages ± S.E.M. Significance was set at *P* < 0.05.

## Results

### Visual discrimination hexagonal swim tank

**Figure 1:**
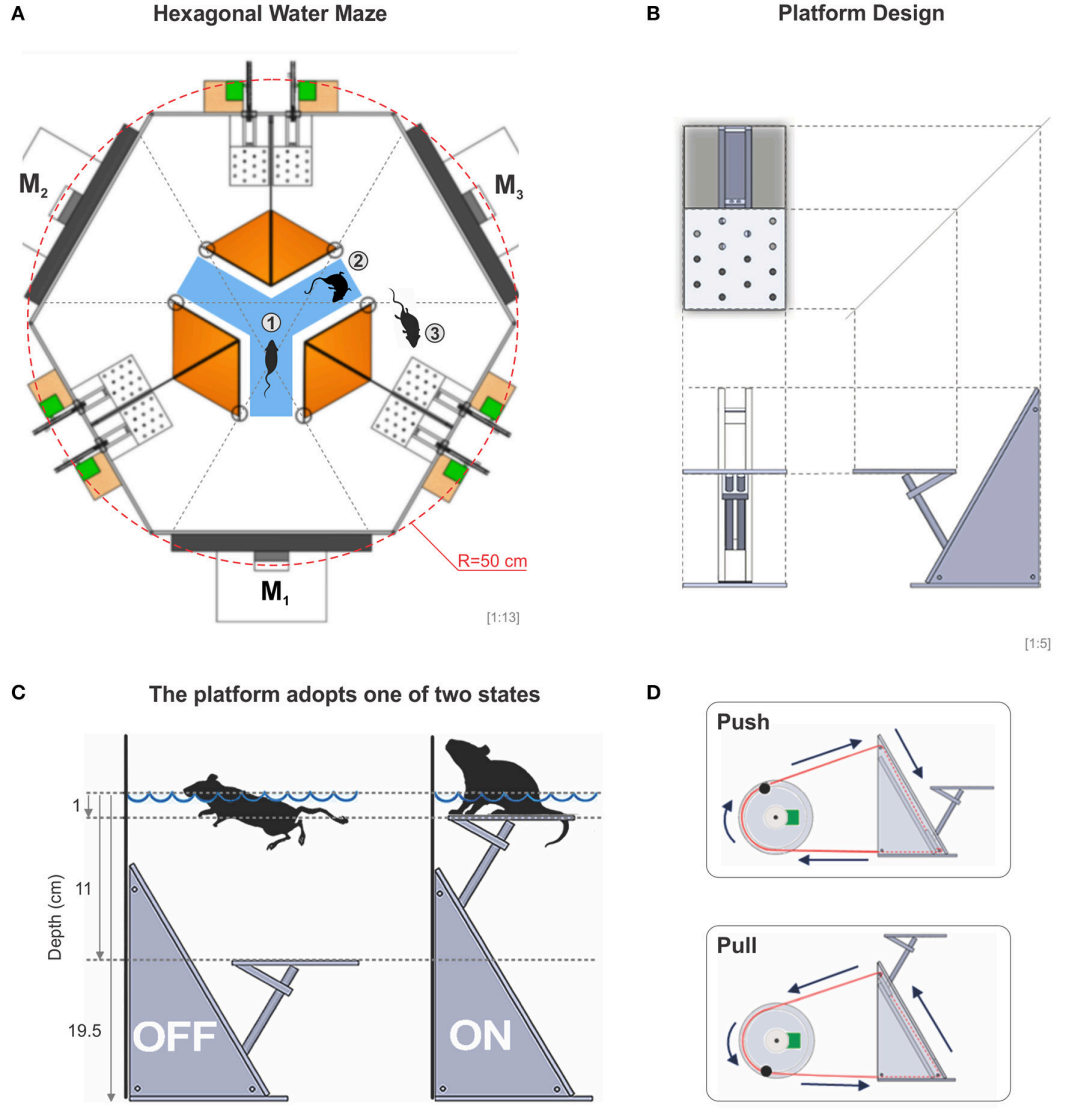
The hexagonal water maze. **(A)** Schematic view of the hexagonal water task. From the decision area (blue), the mice have visual access to three monitors (M_1_, M_2_, M_3_), whose projections can be seen through the glass walls of the pool. In an experiment, the mouse is placed onto a randomly elevated platform and once it submerges, the mouse can enter the decision area from where it has visual access to each of the three monitors, one at a time (1), it can swim around within the decision area (2), or it can enter a chosen arm in search for one of two elevated escape platforms inside the correct arm (3) (total of 6 platforms in the pool). **(B)** Diagram of an orthogonal projection of the platform. **(C)** The platform can be either submerged or elevated 1 cm below the surface of the water allowing the mice to touch it with the rear legs when swimming over it. **(D)** The transition state of the platforms is excecuted through a computer-controlled push-pull pulley system. A special routine moved each pseudo-randomly chosen servomotor 25% of its dynamic range at a time. With this procedure, sounds and vibrations made by the servomotors cannot be used to solve the task.

Our task is based on the fact that mice are instinctively good swimmers and like to escape from water to a solid substrate, whose position can be predicted by visual cues (Prusky et al., 2000; Trevino et al., 2013). Thus, the apparatus we built consisted of a hexagonally shaped pool of 50 cm per side, filled with tap water (21 ± 1 °C) to a depth of 19.5 cm. Three interior white acrylic dividers extended from the side walls toward the center of the pool, creating an interior decision chamber with access to three identical arms. This arrangement formed a virtual Y-maze inside the hexagon (Figure 1A). At the end of each arm, we placed flat computer screens which displayed, through the glass, a reinforced discriminative (S^D^) on one monitor and non-reinforced (S^Δ^) stimuli on the two remaining monitors. The system had a total of 6 computer-controlled acrylic platforms (8 cm long, 8 cm wide, 18 cm high), two per arm, placed ~25 cm to the left and right sides of the monitors, adjacent to the dividers (Figure 1A). Each platform was controlled independently from the others and could adopt one of two states, either submerged at 11 cm or elevated at 1 cm below the water surface, respectively (Figures 1B,C). Through a pulley, we were able to push or pull a closed-loop system based on nylon wire that submerged or elevated each platform (Figure 1D). We provide a detailed description of how to build the dividers, platforms and drainage system in Supplementary Figures 1–3.

### Automatic control of the task

**Figure 2:**
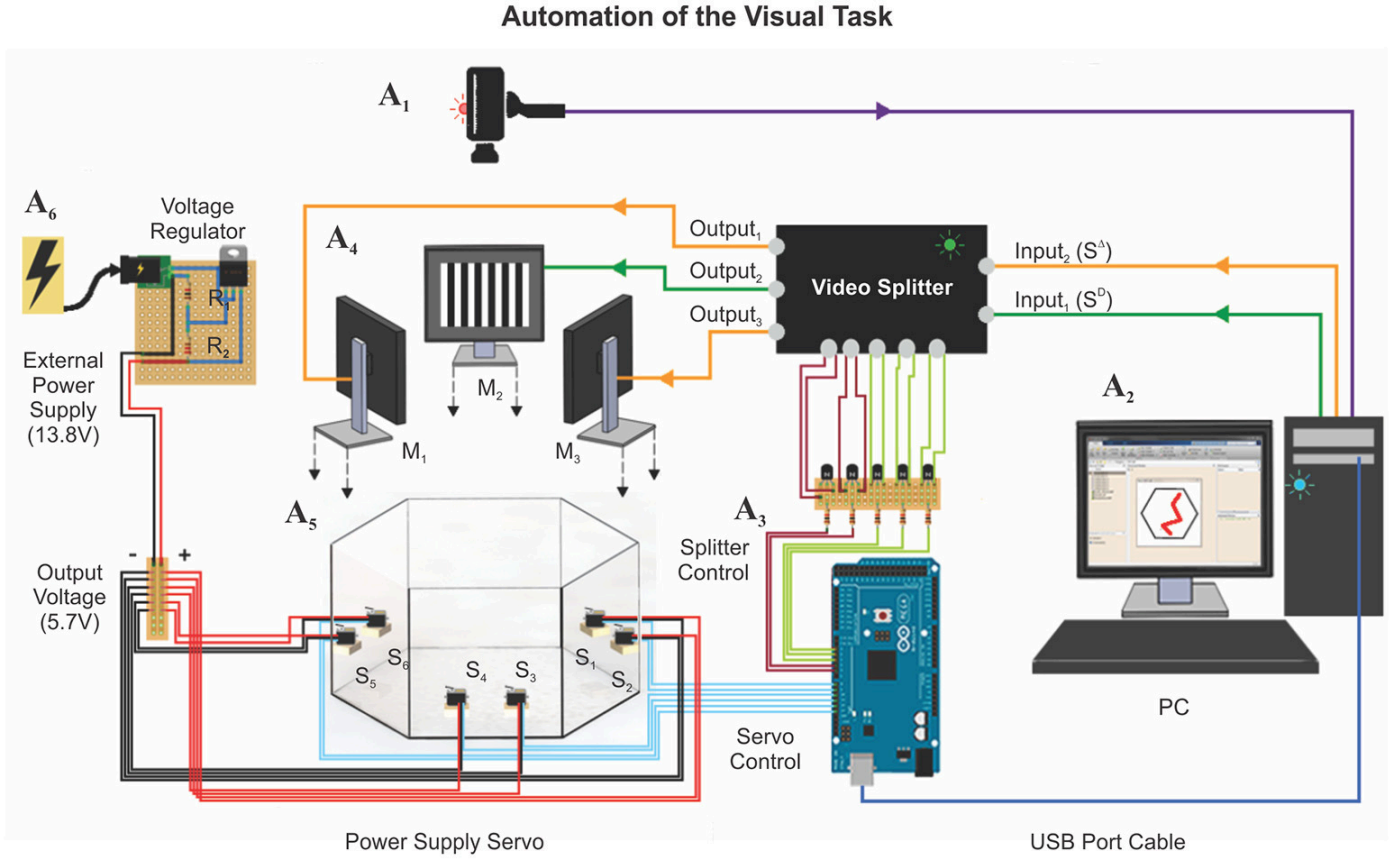
Automation of the visual task. The automation of the hexagonal water maze is based on two core elements: (1) A webcam is mounted on top of the pool (A_1_, see Methods), to trace the position of the animal at ~30 FPS; (2) a computer (A_2_) controlling a video splitter (A_3_) that determines where to project the S^D^/S^Δ^ stimuli (A_4_: M_1−3_) and the six servomotors (A_5_: S_1−6_; 11 Kgf cm) connected to an external power supply (A_6_) that controls the state of the platforms. The tracking system measured several additional behavioral parameters during training: (1) escape latency, (2) trajectory (search path), (3) swim distance traveled, (4) instantaneous and average velocities, (5) swim path error, and (6) proximity to platform.

To achieve the full automation of the hexagonal swim task, we implemented a series of computer-controlled electronic devices (Figure 2). We destined six servomotors (Tower Pro MG996R; 180 Degree Metal Gear Big Torque 11 Kgf cm) to drive the push-pull mechanism of the platforms (Supplementary Videos 1, 2). We powered them up by using an external power supply (STEREN model PRL-3, 13.8 Volts, 5 Amperes), which was operating through a voltage divider circuit to deliver ~6 Volts to each servomotor (current drain of max. 220 mA/servomotor; Supplementary Figure 3). We provided the visual stimulus sequence randomly, with one monitor projecting the S^D^ and the two remainder the S^Δ^. To establish which monitors projected the S^D^ and S^Δ^ stimuli on each trial, we modified a video switch splitter (StarTech 4 × 4 VGA, model ST424MX) by soldering negative-positive-negative (NPN) bipolar transistors (model 2N2222) to the back-side of its buttons. To control the servomotors and the splitter by a computer, we used an Arduino board (Arduino Mega 2560), which operates at 16 MHz with 54 digital and 16 analog input/output pins. We connected the bases of the transistors through 100 Ω resistances to the digital output ports of the Arduino board (5V) and achieved software control by installing the MathWorks® Support Package for Arduino library (see also Supplementary Figure 3). Finally, to monitor the swimming trajectories of the mice in real time, we attached an HD web-camera (Microsoft LifeCam studio) to the ceiling, 190 cm above the bottom of the tank. A reliable software routine running on a computer identified the location of the mouse and plotted the animal's swimming trajectory in real time (Trevino et al., 2013). This program also allowed us to achieve appropriate control of the platforms. We accumulated five frames of “positional evidence” to consider that a mouse had reached a platform. We controlled the functionality of the entire apparatus via software written in MATLAB and synchronized it with the video tracking system, the platforms, and the splitter. We later performed the data analyses on the swimming paths with similar programs.

### Calibration and testing of the apparatus

We implemented two main calibration routines for the pool. The first used the camera on top of the pool to take an overview picture. With this image, we defined a reference system by using polar coordinates to align the exterior and interior walls of the pool, the internal decision chamber, and the position of the six platforms. The second calibration routine allowed the experimenter to define the range with which the servomotors would elevate and submerge each of the platforms. The process began with a fully submerged platform and by gradually pulling the wire, with decreasing angular gradients, the platform progressively, yet smoothly, reached its proper elevation. We stored the angular limits for that platform and repeated the procedure until all the platforms got calibrated.

We then tested the performance of the entire system under conditions of extreme demand. On a first approach, we characterized the time that was required to change the state of all platforms between trials. This interval had an average of 10.87 ± 0.02 s and was stable over the course of thousand repetitions. We also wondered whether the platforms achieved their elevations accurately and found that their performance was stable without any user intervention. Similarly, the video system that tracked the mouse's position operated a stable speed of 31 ± 2 FPS and the servomotors always remained far below a temperature limit of ~55°C, necessary for their proper functioning (Supplementary Figure 4).

### Training visually guided behavior

**Figure 3:**
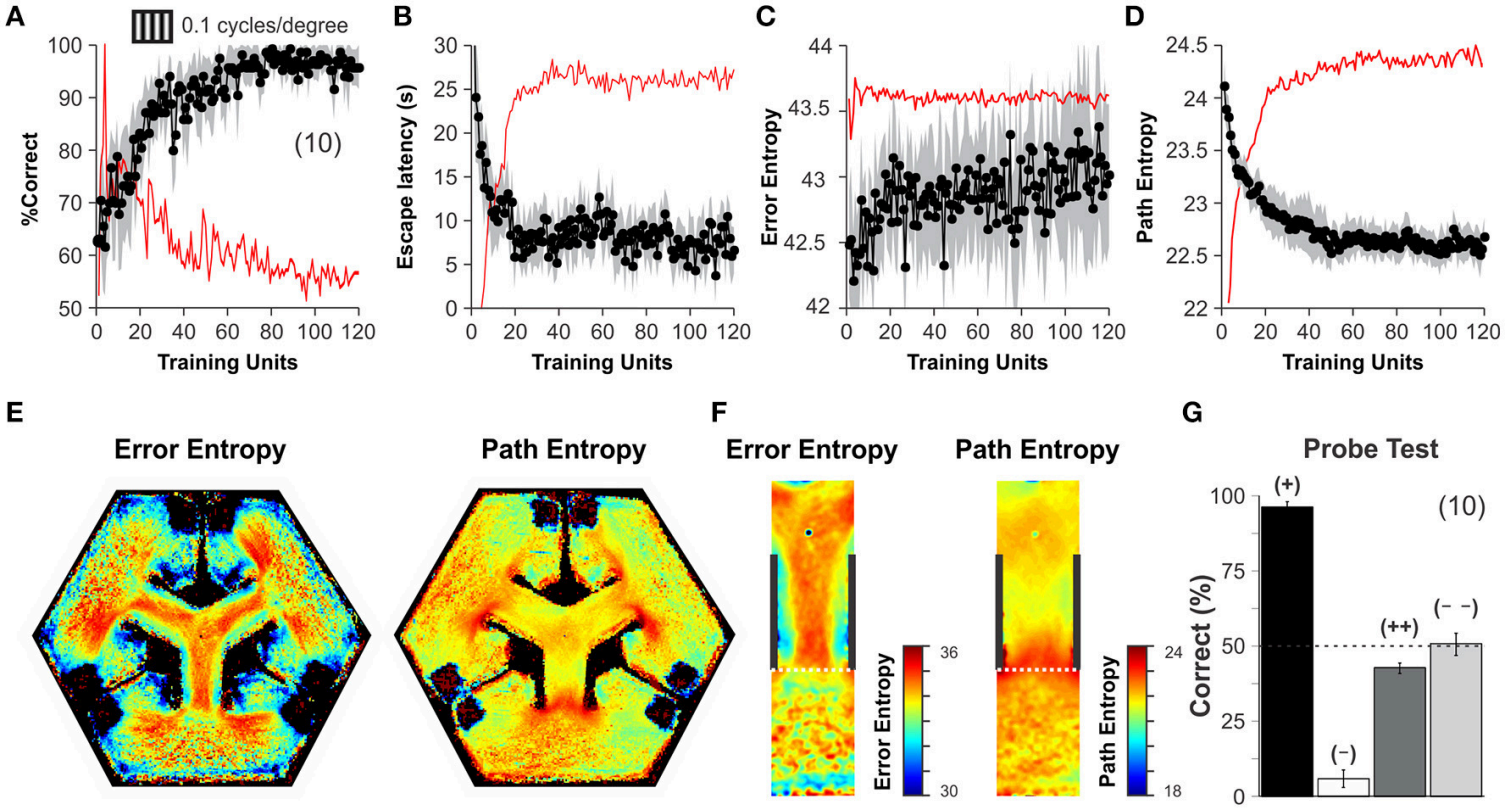
Discrimination learning in the automated hexagonal water maze. The mice familiarized with the swimming pool and the general requirements of the task during an initial week of “pre-training.” **(A)** During their first day of training, the mice performed at ~50% chance level, yet they learned the visual task within 60 training units and discriminated stably thereafter (Wilcoxon test, *P* < 0.001; see Methods). Task acquisition was also paralleled by an asymptotic decrease in escape latencies **(B)**, increase in error entropies **(C)**, and a decrease in path entropies **(D)**. The red lines in panels A-D depict a smoothed version of the first derivative of each dependent variable. Stabilization of the first derivative indicates that the animals reached their asymptote of learning. **(E)** Heterogeneous error (left) and path (right) entropy density maps obtained during training, particularly within the decision area **(F)**. **(G)** A “probe test” with single trials that employed permuted combinations for S^D^ and S^Δ^ stimuli. High accuracy of 96 ± 2% when the platforms were elevated at the S^D^ arm (+, black bar), but 6 ± 3% when elevated in another S^Δ^ arm (–, white bar) (*n* = 10 mice, *t*-test, *P* < 0.001). Chance performance when projecting S^D^ (43 ± 5%, dark gray) or S^Δ^ (51 ± 4%, clear gray) on all monitors (*n* = 10 mice each). These results indicate that visual discrimination of S^D^ from S^Δ^ occurs without contextual cues. Number of mice in parenthesis.

After corroborating the stable operation of our automated system, we applied a training schedule to a group of mice to test its reliability during an experiment. All mice were behaviorally naïve to the task and began the training phase with their correct choices at 50% chance level (Wilcoxon test, *P* = 0.78, *n* = 15), yet they gradually reached very high discrimination performance levels during the next 10 days (Correct choice| before training: 66.40 ± 1.48 %, after training: 97.33 ± 0.53 %, *n* = 10; one-way ANOVA, *F*_(1,17)_ = 14.47, *P* < 0.001, Bonferroni's *post hoc* test, *P* < 0.001; *t*-test, *P* < 0.001; Figure 3A; (Trevino et al., 2012). Both path lengths and escape latencies (the interval required to find and mount the escape platform) decreased asymptotically as learning progressed (escape latency | before training: 20.43 ± 3.69 s, after training: 6.47 ± 0.44 s, *n* = 10; one-way ANOVA, *F*_(1,17)_ = 13.72, *P* < 0.001, Bonferroni's *post hoc* test, *P* < 0.001; *t*-test, *P* < 0.001; Figure 3B). We calculated a proximity measure relative to the escape platform by continuously sampling the position of the mouse and its distance to the escape platform. The swim path error and proximity to the platform served to calculate the error- and path-entropies of the mice during training (see Methods; Maei et al., 2009). The error entropy slightly increased ~1.20% whereas the path entropy decreased ~6.25% during the course of training (Error Entropy | before training: 42.53 ± 0.09, after training: 43.05 ± 0.04 s, *n* = 9; one-way ANOVA, *F*_(1,17)_ = 13.16, *P* < 0.05, Bonferroni's *post hoc* test, *P* < 0.001; *t*-test, *P* < 0.001; Path Entropy | before training: 23.47 ± 0.14, after training: 22.59 ± 0.04 s, *n* = 9; one-way ANOVA, *F*_(1,17)_ = 13.72, *P* < 0.05, Bonferroni's *post hoc* test, *P* < 0.001; *t*-test, *P* < 0.001; Figures 3C,D). The increase in error entropy over training might reflect an adaptation of the swimming strategies within the decision area, whereas the drop in path entropies might reflect more focused swimming paths.

By using all swimming trials from all mice during training, we calculated the 2D frequency distributions for error and path entropies as a function of position within the pool (Figure 3E). These density maps revealed strong inhomogeneities inside the pool, with higher values inside or at the decision area (Figure 3F). We also conducted a “probe test” at the end of training and confirmed that the mice used the visual information from the S^D^/S^Δ^ stimuli to solve the task (Trevino et al., 2012). The procedure involved testing new contingencies in only 10% of randomly chosen trials (to avoid extinction). Task performance was 96.41 ± 1.69% correct when the platforms were elevated in the S^D^ arm, but it fell to 5.78 ± 2.91% when they were on the S^Δ^ arm (*n* = 10 mice, *t*-test, *P* < 0.001). In contrast, discriminative performance was at chance levels of 42.67 ± 1.76 % and 50.67 ± 3.77 % when all monitors displayed either the S^D^ or the S^Δ^ stimulus, respectively (*n* = 10 mice each; Wilcoxon test, *P* > 0.4; Figure 3G). These results demonstrate that visual discrimination performance was under the control of the visual stimuli displayed by the monitors and empirically confirms that chance behavior in the hexagonal pool corresponds to ~50% correct choices.

### Assessment of stereotypical patterns of choice behavior

**Figure 4:**
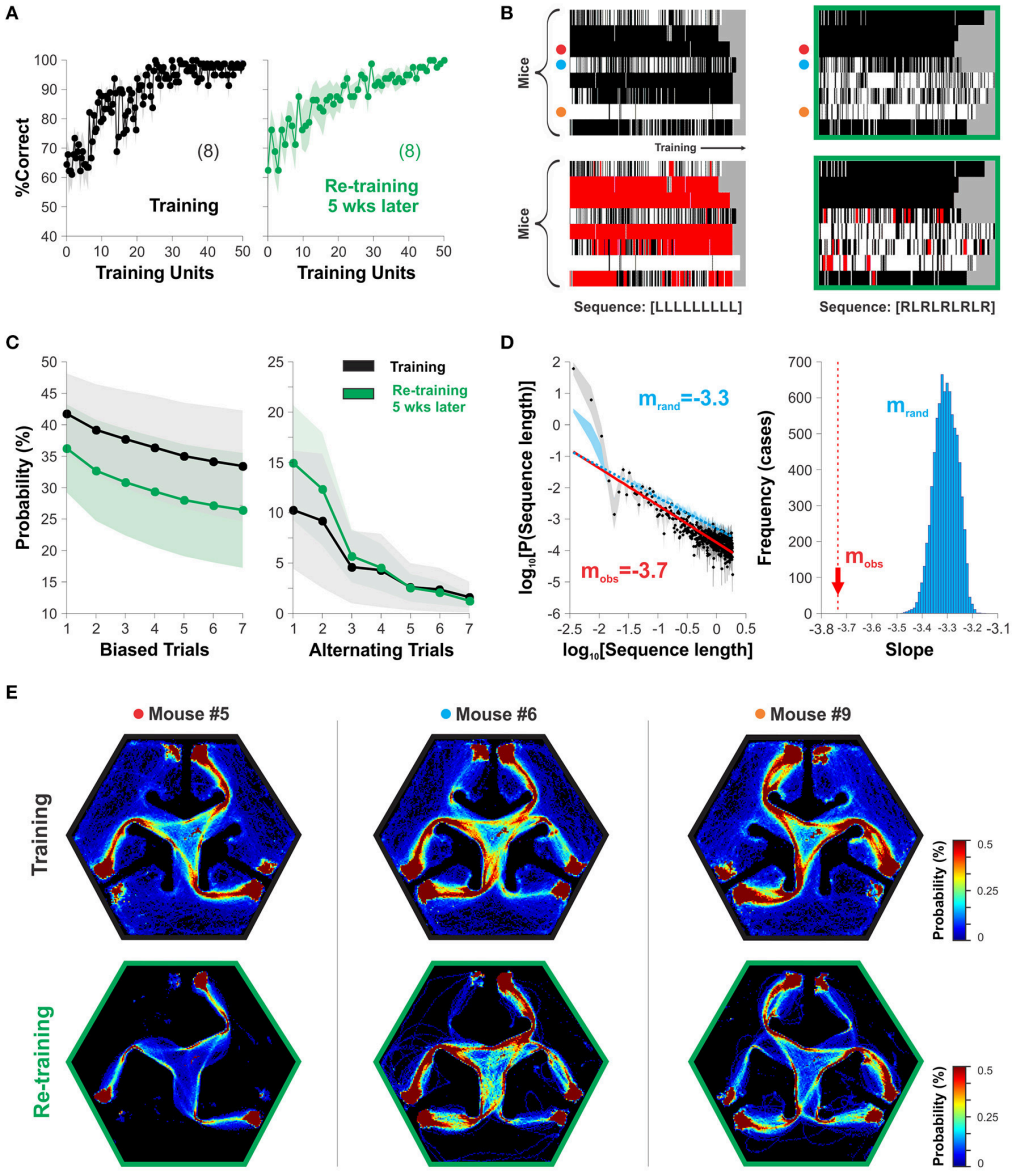
The hexagonal water maze serves to detect stereotyped choice behavior. The mice can adopt visual and non-visual strategies during the discriminative task. **(A)** The %correct choice increased during training (left graph) and re-training (5 weeks later, right graph) phases. **(B)** The panels display the choices of individual mice as rows (y-axis) with either white (right choices) or black (left choices) colors as a function of the training trials (x-axis; Trevino, 2014. Individual stereotyped choices are reflected as horizontal white or black blocks of different lengths. The panels below show two examples of sequence analysis performed on the same choice records. The red squares superimposed on the choice diagrams show the occurrence of such query sequences. Target sequences on the bottom. **(C)** Stereotyped/alternating behavior are stable over time. **(D)** The size distributions (sequence length) for stereotyped choice behavior follow a power law with a significant slope of m_obs_ = −3.74, different to chance levels (m_rand_ = −3.30; 1,000 surrogates). **(E)** Probability distributions for the swimming trajectories of three mice. Number of mice in parenthesis.

Our design of the hexagonal pool had two platforms located symmetrically to the left and right sides of the projected stimulus inside each arm (Figure 1A). This arrangement allowed the mice to find escape from water to both left and right sides of the S^D^, allowing them to freely express their preferred side of swimming without any additional swimming costs. To search for stereotyped swimming behavior in our task, we took choice data from two relevant training epochs during our experiments (Correct choice| training: 97.04 ± 0.73 %, after re-training: 97.61 ± 0.68 %, *n* = x; one-way ANOVA, *F*_(1,17)_ = 1.83, *P* = 0.43; Figure 4A). By inspecting their swimming records, we noticed that the mice displayed different side-sequences during training some of which consisted of repeatedly approaching a platform located on the same side within the chosen arm (Trevino, 2014). To illustrate this stereotyped choice behavior, we labeled and plotted the trials in which the mice swam to the right platform in white, and in black those in which they swam to the left (Figure 4B). We counted the number of blocks of stereotyped (i.e., biased trials) and alternating sequences of different lengths (sequence length) from these groups and estimated their probability of occurrence, respectively (Figure 4C; Trevino, 2014). Notably, the stereotyped and alternating choice behaviors were stable between the two acquisition epochs, separated by 5 weeks from each other (P(Biased trials) RM-ANOVA, *F* = 0.28, *P* = 0.88; P(Alternating trials) RM-ANOVA, *F* = 0.59, *P* = 0.66). The size (sequence length) distributions for stereotyped choice behavior followed a power law with a characteristic slope of −3.74 (the linear part of the plot indicates power law; Figure 4D). We assessed the significance of the slope by comparing it against the slopes obtained from surrogate groups made by random permutations of the side of the mice's choices. We rejected the null hypothesis (i.e., the slope was significant) because all 1,000 surrogate slopes were higher than the empirical one (m_obs_ = −3.7, m_rand_ = −3.31 ± 0.04; Figure 4D). Finally, by inspecting the density maps of the swimming trajectories from three sample mice during training (top row) and 5 weeks later (lower row) we can easily appreciate how choice stereotypies are closely related to motor ones (Figure 4E). These results exemplify how the hexagonal pool can be used to characterize and study stereotyped choice behavior in mice.

### Primary visual cortex is necessary to solve the hexagonal water maze

**Figure 5:**
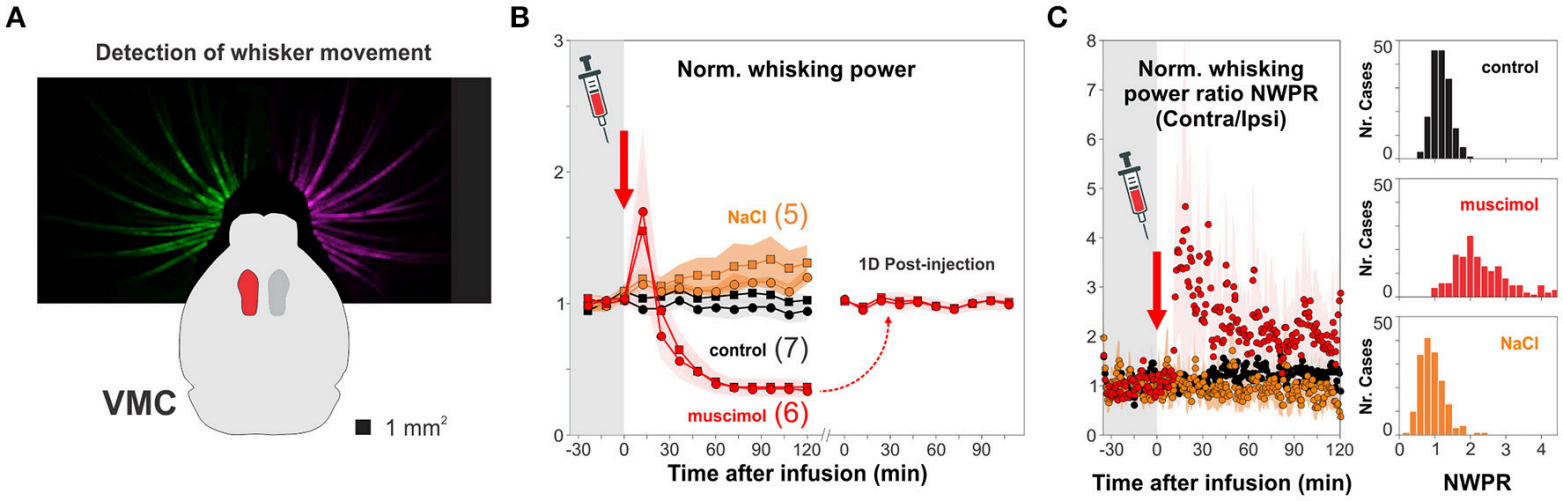
Pharmacological inactivation of brain circuits. **(A)** We chose the vibrissa motor cortex (VMC) to validate the effectiveness of cortical micro-infusion of the a GABA_A_ agonist muscimol (red) by tracking the movement of the entire left and right whisker sets with a camera. **(B)** Unilateral infusion of 1 μl, 25 mM muscimol (red) reversibly reduced >60% the overall movement of left (circles) and right (squares) whisker sets compared to control, uninjected mice (black circles and squares for left and right whiskers, respectively; normalized motility 30 min before infusion; one-way ANOVA, *F*_(2,26)_ = 27.55, *P* < 0.001). **(C)** Unilateral injection of muscimol into VMC deep layers increased contralateral/ipsilateral whisking power (one-way ANOVA, *F*_(2,26)_ = 19.54, *P* < 0.001). No changes in whisker movement when injecting the same volume of 1 μl NaCl 0.9% (orange). The microinjections of muscimol into the VMC did not affect general locomotion. Number of mice in parenthesis.

Visual performance depends on information processing in the retina but also on the intact function of V1 (Prusky et al., 2008; Glickfeld et al., 2013). Therefore, we wanted to know how V1 was involved in solving the hexagonal pool. We developed a micro-injector pump, inspired on a previous design (Wijnen et al., 2014), to pharmacologically inactivate targeted brain circuits (Supplementary Figure 5). We first validated our method by injecting muscimol, a GABA_A_R agonist, into the vibrissa motor cortex (VMC; Figure 5A), a circuit involved in whisker motor control, with active and suppressive actions on free whisker movement (Sreenivasan et al., 2016; Ebbesen et al., 2017). We found that unilateral injection of muscimol, but not of saline solution, reduced >60% the overall motility of left/right whisker systems for more than 2 h after the injection (Figure 5B). The effect of unilateral muscimol injection was asymmetric because the right/left interleaved muscimol infusions produced a ~20% increase in normalized contralateral/ipsilateral whisking power ratio, as previously reported (Ebbesen et al., 2017); Figure 5C). Also, whisker motility was back to normal 1 day after injection (*t*-test, *P* > 0.5; Figure 5B). These results demonstrate that our micro-infusion method can be used to reversibly inactivate cortical circuits.

**Figure 6:**
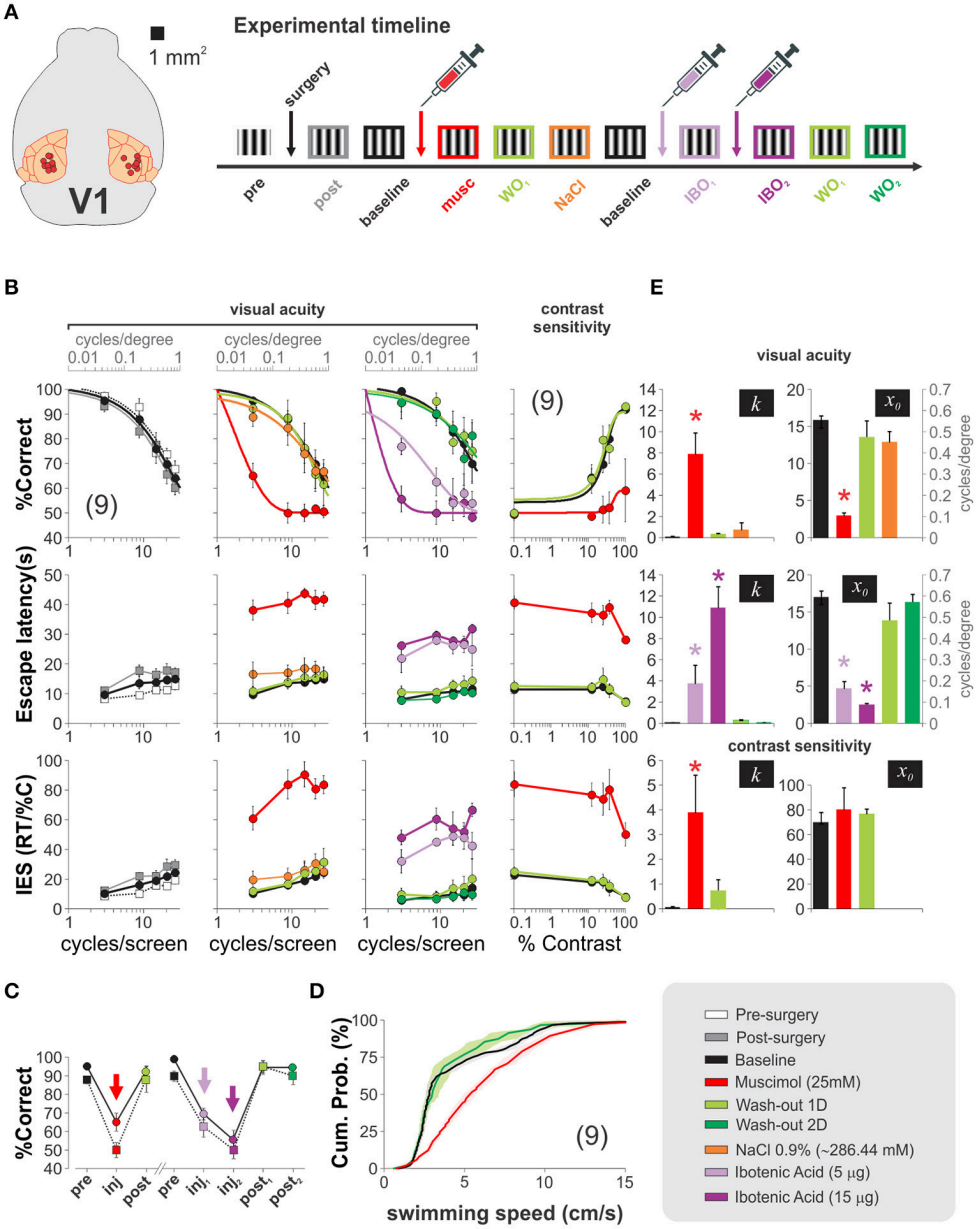
Mouse primary visual cortex is used to solve the hexagonal water maze. **(A)** Diagram depicting implantation sites for nine succesfully cannulated mice and timeline of the experiments. **(B)** Psychometric curves reveal no detrimental changes 4 days after cannulae implantation (pre-surgery: white squares vs. post-surgery: gray squares; baseline: black squares). Bilateral injection of 1 μl, 25 mM muscimol (red circles) into V1 dramatically impaired the visual acuity (2nd column) and contrast sensitivity (4th column) of the mice, but returned to baseline conditions 1 day after infusions (light green circles). In recovered animals, injections of 500 nl (light purple) and 1,500 nl (dark purple) of 10 μg/μl ibotenic acid over two consecutive days reversibly reduced the visual acuity of the mice (one-way ANOVA, *F*_(3,27)_ = 9.49, *P* < 0.05; Wilcoxon test, *P* < 0.01), also illustrated in panel **(C)**. **(D)** Faster swimming speeds for muscimol injected mice against control and recovery, excluding the possibility of impaired motor skills. **(E)** The bar plots illustrate the optimized slopes (*k*) and midpoints (*x*_0_) for the logistic model fits from each experimental condition (one-way ANOVA, *P* < 0.05, all cases). Asterisks represent significative differences. Number of mice in parenthesis.

We then aimed to explore the contribution of V1 processing to the hexagonal pool. To solve the task, the mice had to visualize the S^D^ from a decision point, forcing them to make their choices at a fixed distance from the monitors. This geometry ensured fixed spatial properties of the stimuli from this viewing point. We characterized the psychometric curves of a group of trained mice by using static gratings with variable spatial frequencies (Figure 6B). There were clear behavioral changes as a function of the spatial frequency of the stimulus. At low spatial frequencies, the mice swam directly toward the gratings, whereas as the spatial frequencies increased and approached the mice' visual acuity threshold, the animals took more time to make their choices (i.e., longer escape latencies). We averaged the proportion of correct choices for all tested mice as a function of spatial frequency and fitted a logistic function to the data (continuous lines, Figure 6B). From these fits, we extracted a spatial resolution threshold of 0.50 ± 0.02 cycles/degree of visual angle (*n* = 9; see Methods). This resolution is consistent with previous reports (Prusky et al., 2000; Wong and Brown, 2006; Trevino et al., 2012). Similarly, to quantify the contrast thresholds, we selected a stimulus with a low spatial frequency that the mice could reliably solve at high contrast (3 cycles/screen = 0.1 cycles/degree), and tested it with permuted lower contrasts (5 contrast levels tested; fourth column of panels in Figure 6B, see Methods). Once again, we fitted a logistic function to the data to determine a threshold of 28.06 ± 2.28% contrast, similar to characterizations from other researchers (Glickfeld et al., 2013). These results corroborate that the spatial frequency and contrast of the stimuli determined the visual performance, the escape latency and the inverse efficiency score (IES) of the mice (the IES is the ratio of the escape latencies divided by the proportion of correct choices, a metric aimed to summarize a possible accuracy/speed trade-off). Our next step was to test how inactivating V1 transformed these psychometric curves. We depict an outline of the experiment on the right side of Figure 6A. We successfully implanted bilateral cannulae in V1 in 9/10 trained animals, allowed them to recover, and re-trained them until their visual performance returned to baseline (% correct, *F* = 1.37, *P* = 0.22; escape latency: *F* = 2.92, *P* < 0.01; inverse efficiency score: *F* = 1.76, *P* = 0.09; 3 groups 5 w. measurements per group, repeated measures ANOVA tests). Next, we bilaterally infused V1 and found that injection of muscimol, but not vehicle, produced a left-ward shift of the psychometric curves, an increase in escape latencies and inverse efficiency scores with respect to control conditions (% correct, *F* = 2.27, *P* = 0.01; escape latency: *F* = 0.84, *P* = 0.60; inverse efficiency score: *F* = 1.42, *P* = 0.17; *n* = 4 groups with 5 measurements per group, repeated measures ANOVA tests). This effect was paralleled by a dramatic decrease of ~86% of the visual acuity of the mice (baseline: 0.50 ± 0.02 cycles/degree; muscimol: 0.07 ± 0.01 cycles/degree, *n* = 9). Interestingly, however, choice performance after muscimol injection was not at 50% chance level when using the low spatial frequency stimulus of 3 cycles/screen (*t*-test, *P* = 0.01). Muscimol injections were also effective in producing a two-fold reduction of the contrast sensitivity of the mice to 60.47 ± 8.38% contrast (*n* = 9; % correct, *F* = 3.01, *P* < 0.001; escape latency: *F* = 1.21, *P* = 0.31; inverse efficiency score: *F* = 0.86, *P* = 0.55; 5 groups w. 5 measurements per group, repeated measures ANOVA tests; (Glickfeld et al., 2013).

We additionally tested the effect of injecting ibotenic acid into V1 of fully recovered mice. We found a graded detrimental effect in the psychometric curves by injecting the drug (two consecutive days: 5 μg first injection, 15 μg second injection; %correct, *F* = 1.32, *P* = 0.21; escape latency: *F* = 1.20, *P* = 0.29; inverse efficiency score: *F* = 0.88, *P* = 0.58; 5 groups with 5 measurements per group, repeated measures ANOVA tests). The visual acuities were also reduced (baseline: 0.50 ± 0.02 cycles/degree, ibotenic acid 5 μg: 0.15 ± 0.03 cycles/degree; ibotenic acid 15 μg: 0.06 ± 0.01 cycles/degree). These results indicate that the pharmacological manipulations of V1 profoundly impaired the visual acuity and contrast sensitivities of the mice (Figures 6B,C).

Although escape latencies were bigger after the inactivation's, we found that this increase was most often the result of the mice swimming across the pool several times near the decision area while inspecting the screens before making their choices (Prusky et al., 2000). Indeed, an analysis on the average swimming velocities of the mice per trial revealed that the muscimol injected animals actually swam ~28% faster than the control ones (pre-surgery: 4.90 ± 0.19 cm/s; muscimol: 6.28 ± 0.20 cm/s; wash-out: 4.76 ± 0.34 cm/s; *P* < 0.001; Kolmogorov-Smirnov test; Figure 6D). So, the drop in visual performance cannot be explained by a reduction in motor capacities. In Figure 6E, we illustrate the effects that the intra-cerebral injections had on the optimized parameters extracted from the psychometric fits. These results demonstrate that the primary visual cortex processes visual information required to solve the hexagonal pool.

## Discussion

We developed a fully automatic apparatus that allows visual training and testing of adult mice with minimal experimenter intervention. The system implements a two-alternative forced-choice (2AFC) task inside a hexagonal pool. The apparatus includes a fast video tracking system and computer-controlled platforms which allows a single person to train multiple mice, with each mouse performing up to 70 trials per day. Other automated systems have been developed to test visual discrimination capacities of mice. They are based on food or water deprivation schemes and usually require ~16–22 days to reach 80% correct choice behavior (Bussey et al., 2008; Busse et al., 2011; Yu et al., 2018). In contrast, our aquatic task is based on the fact that mice are highly motivated to escape from water, requiring ~13 days of pretraining plus training to reach 95% of correct choices. Clearly, water and dry mazes should involve different motivational and learning mechanisms which were not addressed here (see v.gr. Ormerod and Beninger, 2002).

We developed analytical tools to quantify relevant behavioral features like correct and stereotyped choices, path lengths, escape latencies, swimming speeds, and also to characterize more complex adaptive strategies during acquisition. A distinctive feature of our task is that we placed two escape platforms (US, for unconditioned stimuli) equidistant from the monitor that projected the S^D^ in each arm. This geometry allowed the mice to swim either to the left or right side of the S^D^ to receive a –delayed– reward when reaching the US. It was precisely this setting that allowed us to use the hexagonal pool to assess the stereotyped choices of the mice. In this condition, stereotypies are independent of discriminative choices because the expression of swimming to either platform has no differential impact on accessing the US. We found robust stereotyped behavior over the course of several weeks, revealing that this behavior is stable and consistent across mice (Busse et al., 2011; Trevino, 2014). Also, suggesting the existence of a power law, we found a linear relationship between the probability of observing an array of biased choices and the sequence length (Bak et al., 1987; Hanel et al., 2017). Another important property of stereotyped behavior is that it is strongly dependent on the choice history as this could offer better adaptive strategies to the inherent imbalances in the natural world (Busse et al., 2011; Trevino, 2014; Akrami et al., 2018). The emergence of such biases could be driven by the lack of discriminative information (Killeen et al., 2018), but they could also reflect internal imbalanced processes of individuals (Trevino, 2014).

Some authors have documented that smaller distances between S^D^-US tend to evoke more approach to the S^D^ (sign-tracking) than to the US (goal-tracking) whereas goal-tracking is observed at larger distances (Holland, 1980; Silva et al., 1992). Similarly, when the S^D^-US interval is increased (as it occurs in trace conditioning), a reduction in conditioning is observed as it takes more trials for the conditioned response (CR) to be observed and the CR strength is often reduced. These and other observations illustrate how contiguity manipulations influence the CR and how animals are well capable of learning about the relationship between cues and outcomes over many hours and days (Balsam et al., 2010). Certainly, the capacity to associate two separated stimuli is vital for animals as the perceived features of objects are continually changing with time and location. In our experiments, we found that during the initial phases of training, the mice tended to swim more toward the S^D^ followed by an approach to the US. However, once they achieved a high discrimination performance, the animals used the S^D^ very briefly to predict the presence of reward, and left the decision zone to swim directly toward the US (goal-tracking). Thus, non-consumatory S^D^-specific behavior was observed within the decision area, whereas goal-tracking behavior was independent of CS characteristics (v.gr. S^D^ stimuli with different contrasts) and occurred after the animals left the decision area.

The learning of the hexagonal task by the mice depended on many factors. A recent study explored the predictive power of several continuous behavioral parameters to characterize rodent search strategies. The study adapted the concept of entropy—the degree of uncertainty associated with the swimming trajectory—as a performance metric (Maei et al., 2009). We implemented this analysis to take advantage of the richness of the mice swimming trajectory data. The approximation is valid because the hexagonal pool is rotationally symmetric and the positions of the mice and platforms changed pseudo-randomly in a balanced fashion. Accordingly, both the distance from the platform and the distance from the focus of the search path were normally distributed. Using this metric, we found that the mice decreased their path entropies (transition into a more ordered state of entropy) but increased their error entropies, probably due to the emergence of more robust search strategies within the decision area. These entropy measures will serve in future studies to detect subtle differences in search strategies in the hexagonal water task.

Using the automated hexagonal maze, we also characterized the visual acuities and contrast sensitivities of a group of mice (Prusky et al., 2000; Robinson et al., 2001; Wong and Brown, 2006; Trevino et al., 2012; Glickfeld et al., 2013). We conducted each of these measurements within a single day of testing. We made pharmacological micro-infusions to perturb cortical function and found that inactivating V1 impaired the visual conditioned responses at low/intermediate contrasts and at high spatial frequencies, similar to what other authors have shown (Glickfeld et al., 2013). Notably, the behavioral effects of both muscimol and ibotenic acid micro-infusions into V1 did not fully abolish visual function at low spatial frequencies, and were reversible 1 day after injection. This probably reflects a sub-lethal exposure to the latter toxin (Schwarcz et al., 1979; Newsome et al., 1985; Page et al., 1991). Visual inputs from the retina project to the lateral geniculate nucleus (LGN) and the superior colliculus (SC), and these two pathways are known to support the processing of visual information. Indeed, the LGN sends information directly from the optic tract to V1. But also the “extra-geniculate” pathway composed by the SC and the LPN/pulvinar nucleus of the thalamus sends axons and visual information to higher visual areas (Wang and Burkhalter, 2007; Tohmi et al., 2014; Ahmadlou et al., 2017). This second sub-cortical SC pathway might explain the remainder of non-random visual behaviors observed after V1 inactivation. In fact, several studies have shown that many visual capacities, including pattern and form discrimination, are preserved in adult mice after V1 inactivation/destruction (Prusky and Douglas, 2004; Prusky et al., 2008; Tohmi et al., 2014). Therefore, our results indicate that V1 is necessary, but not sufficient, to solve the hexagonal water maze (Otchy et al., 2015). They strongly support the notion that V1 specializes in processing visual information with the highest contrasts and spatial frequencies (Prusky and Douglas, 2004; Glickfeld et al., 2013).

Altogether, the hexagonal water maze constitutes a reliable system to measure visual capacities and perceptual thresholds of mice. The automated task allows longitudinal experiments to search for changes in visual function derived from controlled alterations of visual experience, damage to the visual circuit, and pharmacological and/or genetic manipulations. It constitutes a novel experimental assay to quantify stereotyped behavior and study its potential link to the underlying physiological properties and imbalances of individuals.

## Author contributions

MT: conceived the project, designed and built all devices, performed experiments and histology, analyzed data, made figures, wrote the manuscript; EF: designed and built platforms and micro-injector pump, made figures; CS: performed surgeries and micro-infusions; EL: performed experiments and histology.

### Conflict of interest statement

The authors declare that the research was conducted in the absence of any commercial or financial relationships that could be construed as a potential conflict of interest.

## Supplementary material

Supplementary Figure 1 Assembly of arm dividers. Orthogonal **(A)** and isometric **(B)** projections of the divider with water level depicted as a blue line in panel **(B)**. Using trigonometry, we defined the height and angles of the divider walls to prevent the mice from having visual access to more than one monitor at a time. **(C)** Each divider included a pair of tilted triangular walls which precluded the mice from holding themselves against the internal walls of the dividers. They also included cylindrical “anti-grabbers” on the edges of the walls to prevent the mice from holding themselves onto these walls. **(D)** Isometric scheme on how to assemble all the pieces of the divider. All dimensions in millimeters. Scales in gray brackets.

Supplementary Figure 2 Assembly of platforms. **(A)** The construction of each platform required a total of 16 laser-cut pieces from 3 to 5 mm thick acrylic sheets. The angular perforations in the slider were made with a column drill. The griddle of the platform had a series of aligned perforations to decrease the resistance against the water when moving the platform inside the water. **(B)** The platforms can be easily assembled and bonded **(C)** using Weld-On® 16™. All dimensions in millimeters. Scales in gray brackets.

Supplementary Figure 3 Assembly of additional components of the hexagonal water maze. **(A)** To control the video-splitter (StarTech 4 × 4 VGA Matrix Video Switch Splitter, model ST424MX) with a computer, we used 2N2222 transistors connected with a 100Ω resistance between its base (midline leg) and the digital pins of an Arduino board. The metal-teeth, large-torque servomotors (Servomotor Tower Pro MG995R or MG996R, 11 Kgf cm) were controlled directly from the digital output pins of the Arduino board. **(B)** The servomotors optimally operate between 4.8 and 7.2 Volts. We made a voltage divider since we had a power supply that delivers 13.8 V. The divider is a passive linear circuit that produces an output voltage (V_out_ ≈ 6 V) that is a fraction of its input voltage (V_in_ ≈ 13.8 V). The circuit required an adjustable positive voltage regulator (LM317, STMicroelectronics) and two resistors (R_1_ = 470Ω and R_2_ = 1,800Ω). We welded all electronic components to cupper bakelite. **(C)** The water maze also included a drainage system using a 0.5 HP water pump (Evans, BP1ME050) which drained the pool at 32 l/min. This facilitated the cleaning of the entire system.

Supplementary Figure 4 Reliable performance of the automated task. Stable performance of the automatic training system: **(A)** intervals required to make a state transition of the platforms (Interval, repetition_1−10_: 10.87 ± 0.02 s, repetition|_990−1,000_: 10.86 ± 0.02 s; one-way ANOVA, *F*_(1,17)_ = 0.14, Wilcoxon test, *P* = 0.73; *t*-test, *P* = 0.36). **(B)** Elevation of the platforms (Normalized amplitude, repetition|_1−10_: 100.17 ± 0.11 %, repetition|_990−1,000_: 99.85 ± 0.09 %; one-way ANOVA, *F*_(1, 9)_ = 3.12, Wilcoxon test, *P* = 0.08; *t*-test, *P* = 0.11). **(C)** Speed of the video camera tracking system (FPS, repetition|_1−10_: 30.62 ± 1.72, repetition|_991−1,000_: 31.49 ± 2.06; one-way ANOVA, *F*_(1,17)_ = 0.82, Wilcoxon test, *P* = 0.38; *t*-test, *P* = 0.39) during 1,000 repetitions. (D) The temperature of active or inactive servo-motors connected to their power supply remained well below their limit of ~55°C within 8 hrs of use (without activity: T|_1−10_: 19.81 ± 0.02°C, T|_991−1,000_: 28.97 ± 0.01°C; active servomotors: |T_1−10_: 20.15 ± 0.08°C, |T_991−1,000_: 27.38 ± 0.01°C; one-way ANOVA, *F*_(1,17)_ = 14.47, Wilcoxon test, *P* = 0.01; *t*-test, *P* < 0.001).

Supplementary Figure 5 Micro-injector pump. **(A)** Schematic of the micro-injector pump designed and built in our lab to micro-infuse small volumes (0-1,000 nl) of pharmacological agents at slow speeds of 0.1 μl/min (~1.67 nl/step) directly into the mice's brain. The device controls a stepper motor (NEMA 17HS8401 78 Oz-in) through a high-current motor driver carrier (DRV8825) connected to an Arduino board and requires an external power input (12 V, 1.8 A). The system is mounted on a plastic chassis of 6 mm thick acrylic sheets. When triggered, a clockwise/anticlockwise step in the motor, and a rotation of the threaded bar started pushing forward/backward the plunger holder attached to an adjustable holder for two Hamilton syringes of 10 μl each (Hamilton, 701LT; inner barrel of 0.019 inches; Supplementary Videos 3, 4). Polyethylene tubing connects the syringes with the infusion cannulas. **(B)** Guides and cannulas made with hypodermic stainless steel. The tip of the cannulae extended 300 μm below the guides.

Supplementary Video 1 Animation of the platform's push-pull mechanism inside the hexagonal water maze. The video shows how a platform inside the pool transits from a submerged to an elevated state.

Supplementary Video 2 Side-view of a platform inside the hexagonal water maze. The animation illustrates an orthogonal view of a platform being elevated inside the water maze.

Supplementary Video 3 Moving mechanism of the micro-injector pump. The video displays how the rotation of the stepper motor is transformed into a linear displacement by the micro-injector pump by using a threaded bar.

Supplementary Video 4 Infusion mechanism of the micro-injector pump. Controlled micro-infusion of drugs (or vehicle solutions) by advancing the plunger of two 10μl Hamilton syringes intended for bilateral infusions.
