# Supplementary material for: An Automated Water Task to Test Visual Discrimination Performance, Adaptive Strategies and Stereotyped Choices in Freely Moving Mice
Source: Front Behav Neurosci. 2018 Nov 8;12:251. doi: 10.3389/fnbeh.2018.00251 (PMC6235986; doi:10.3389/fnbeh.2018.00251)

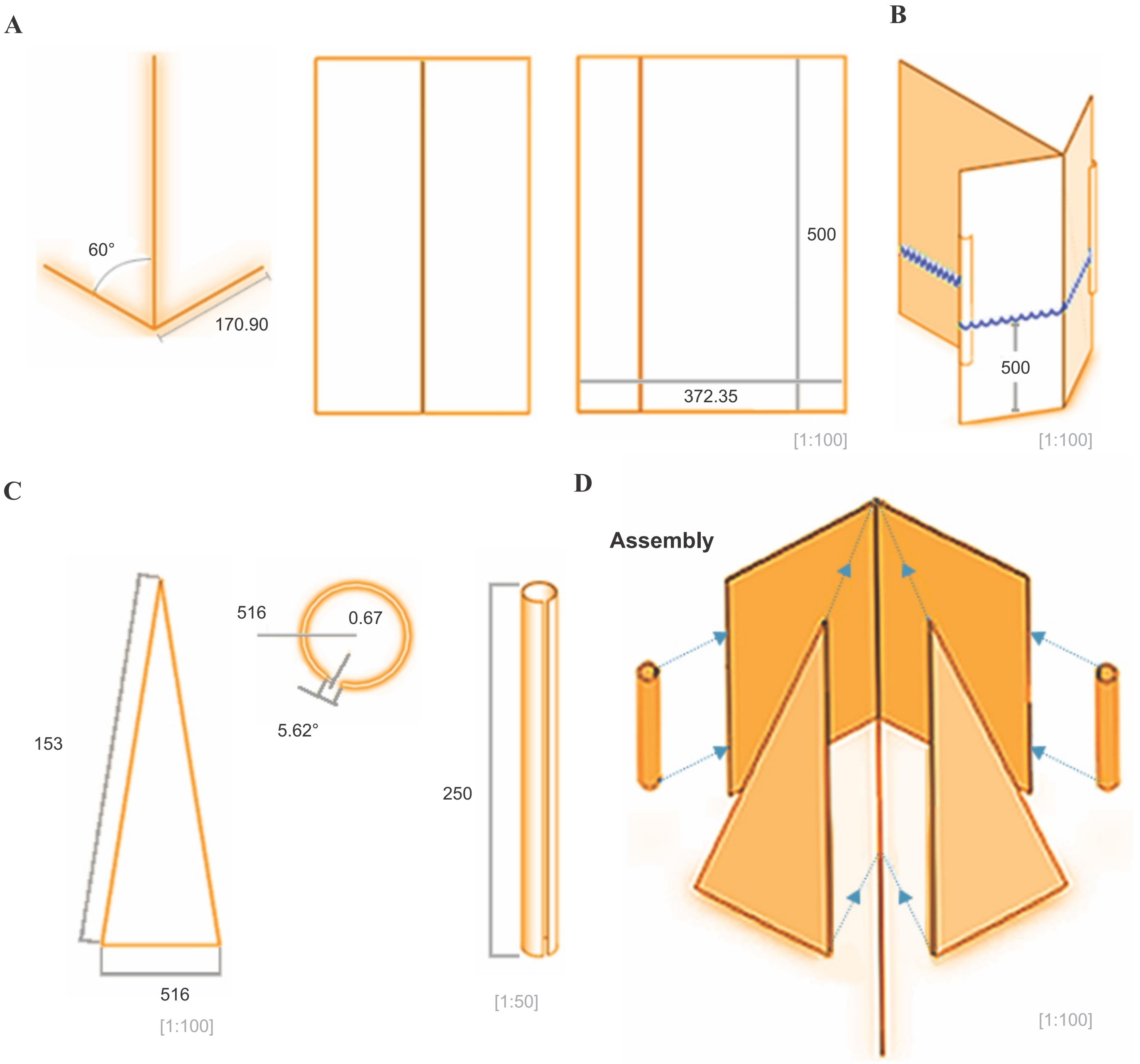

Supplementary Figure 1

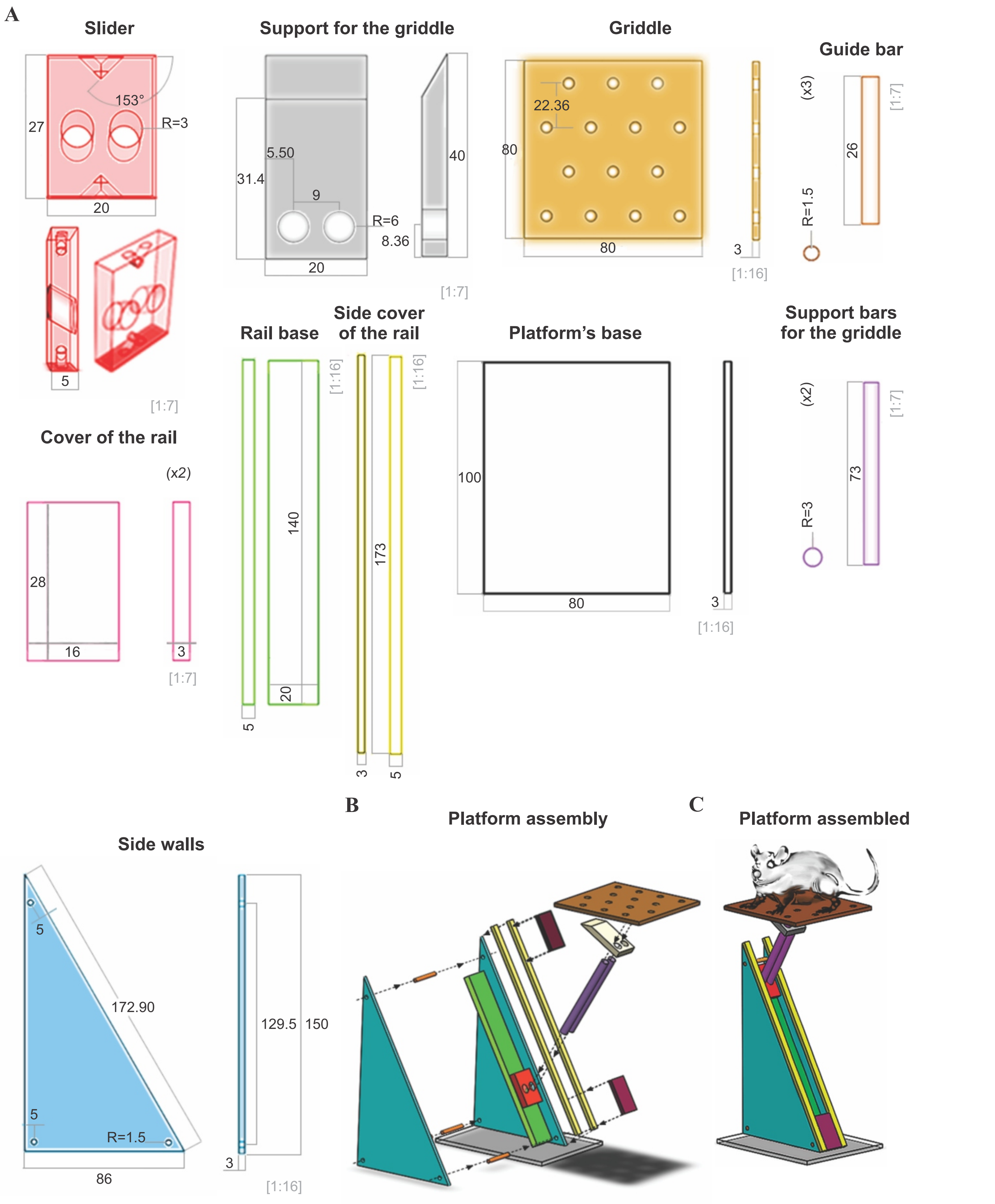

Supplementary Figure 2

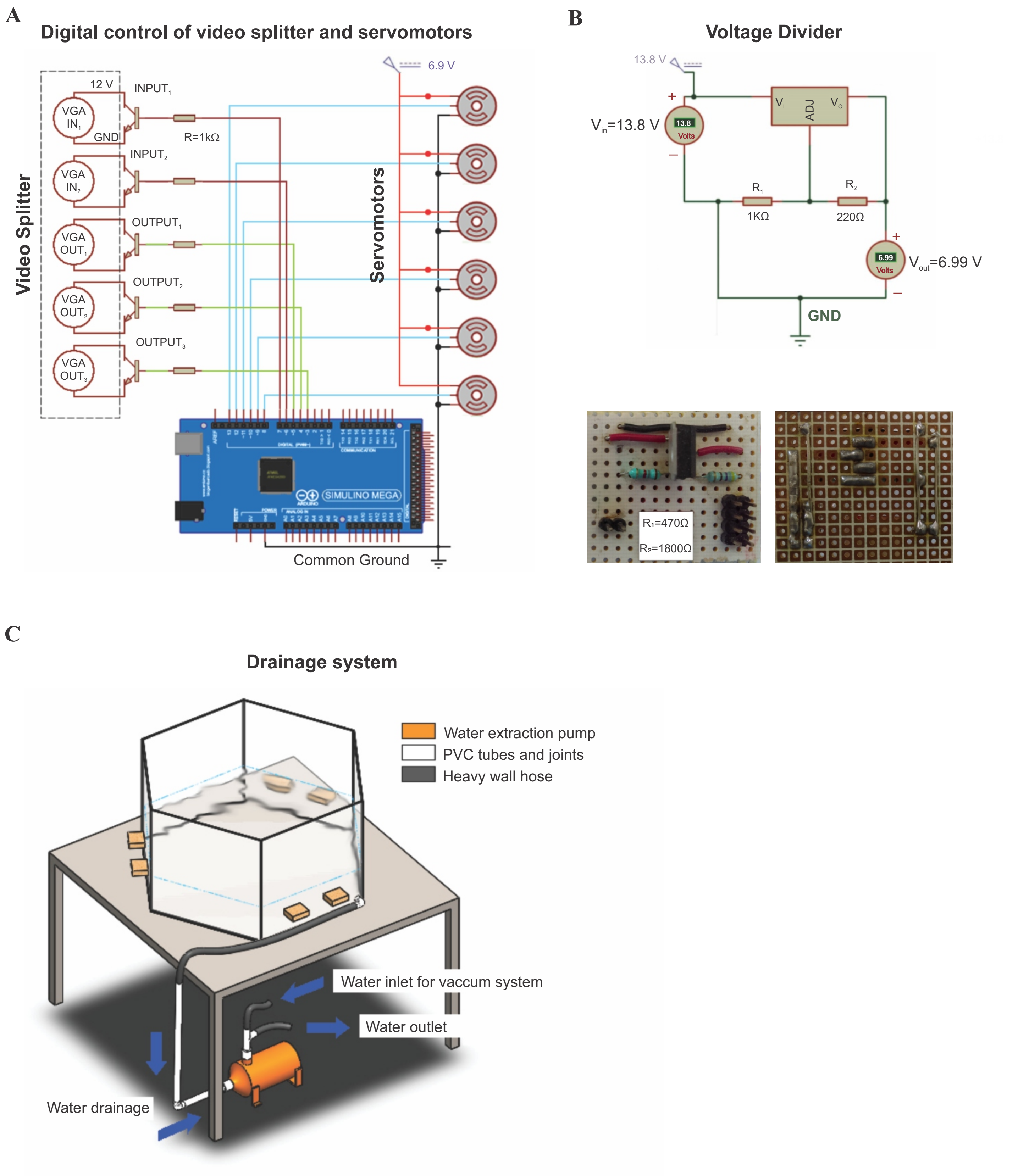

Supplementary Figure 3

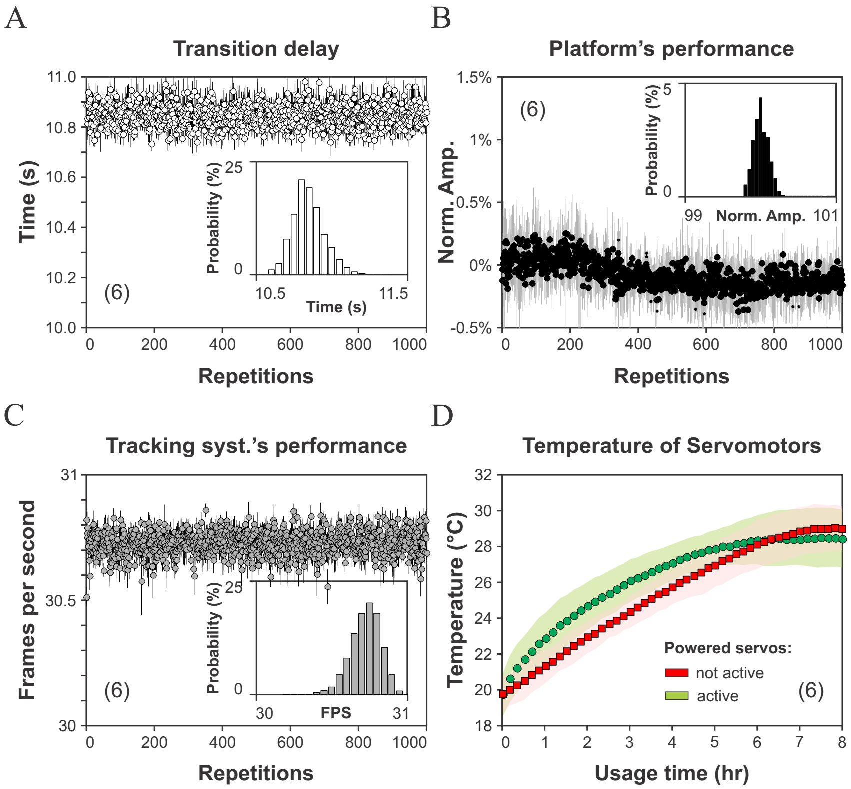

Supplementary Figure 4

A

Computer-controlled micro-injector pump

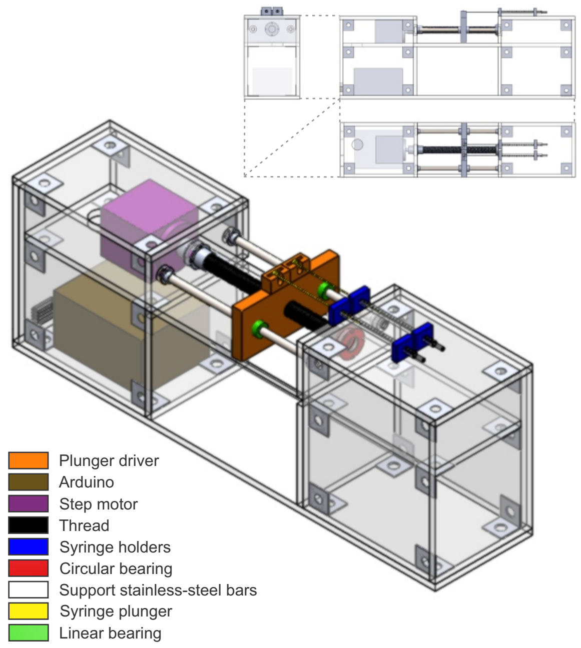

B

Guide and Cannula for V1

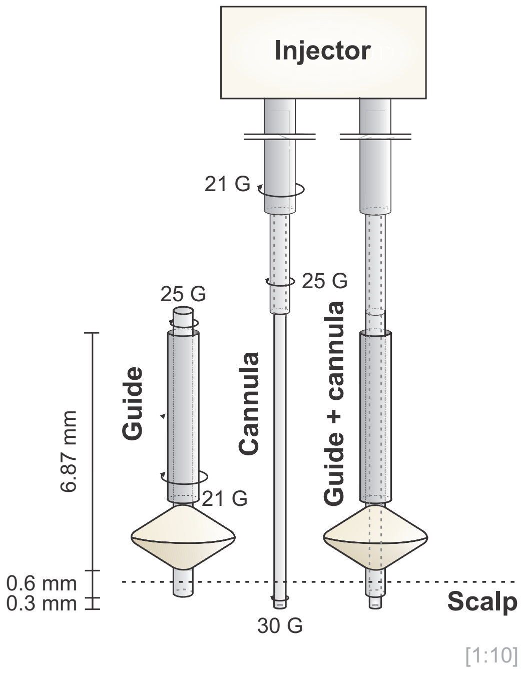

Supplement: Supplementary Figure 1 — Assembly of arm dividers. Orthogonal (A) and isometric (B) projections of the divider with water level depicted as a blue line in panel (B). Using trigonometry, we defined the height and angles of the divider walls to prevent the mice from having visual access to more than one monitor at a time. (C) Each divider included a pair of tilted triangular walls which precluded the mice from holding themselves against the internal walls of the dividers. They also included cylindrical “anti-grabbers” on the edges of the walls to prevent the mice from holding themselves onto these walls. (D) Isometric scheme on how to assemble all the pieces of the divider. All dimensions in millimeters. Scales in gray brackets. [file Image_1.pdf]
